# Unveiling the Role of Endometrial CD-138: A Comprehensive Review on Its Significance in Infertility and Early Pregnancy

**DOI:** 10.7759/cureus.54782

**Published:** 2024-02-23

**Authors:** Prachi A Ughade, Deepti Shrivastava

**Affiliations:** 1 Obstetrics and Gynaecology, Jawaharlal Nehru Medical College, Datta Meghe Institute of Higher Education & Research, Wardha, IND

**Keywords:** diagnostic marker, reproductive medicine, early pregnancy, infertility, cd-138 (syndecan-1), endometrium

## Abstract

This review comprehensively examines the role of endometrial CD-138 (syndecan-1) in the context of infertility and early pregnancy. The endometrium, a dynamic tissue responsive to hormonal cues, plays a central role in fertility, and understanding the molecular intricacies governing its function is crucial. CD-138, a cell surface proteoglycan, emerges as a critical player expressed by various endometrial cell types. Our exploration encompasses a brief overview of the endometrium, introducing CD-138 as a significant molecular entity. The rationale for the review underscores the importance of elucidating endometrial factors in fertility and addresses existing knowledge gaps related to CD-138. Throughout the review, we unravel the multifaceted nature of CD-138 and its involvement in infertility, highlighting its potential as a diagnostic marker. Furthermore, insights into CD-138's role during early pregnancy, including trophoblast-endothelial interactions, are discussed. In conclusion, the findings underscore the clinical implications of CD-138, suggesting its utility in diagnostics and offering prospects for targeted therapeutic interventions. The identified knowledge gaps propel future research directions, promising to deepen our understanding of this enigmatic molecule and its transformative potential in reproductive medicine.

## Introduction and background

The endometrium, the inner lining of the uterus, plays a pivotal role in the complex fertility processes and early pregnancy. Its dynamic nature is finely orchestrated through a tightly regulated interplay of hormonal signals, cellular interactions, and molecular events. Within this intricate landscape, CD-138, also known as syndecan-1, emerges as a noteworthy player, exerting its influence on various aspects of endometrial function [1]. The endometrium, a dynamic tissue, undergoes cyclic changes in response to the menstrual cycle. Primarily composed of epithelial and stromal cells, this lining is characterized by its susceptibility to hormonal fluctuations, orchestrating events crucial for embryo implantation and early pregnancy establishment. Understanding the nuances of endometrial physiology is imperative for unraveling the mysteries surrounding fertility and pregnancy [2].

CD-138, a cell surface proteoglycan, is an integral component of the extracellular matrix within the endometrium. It is expressed by various cell types, including epithelial and stromal cells, and has been implicated in numerous cellular processes, ranging from cell adhesion to signaling. Its multifaceted nature suggests a critical role in the finely tuned orchestration of events within the endometrial microenvironment [3]. The intricate interplay between the endometrium and fertility is underscored by the fact that successful implantation is contingent upon the receptivity of the endometrial lining. The endometrium undergoes cyclic changes, preparing for embryo implantation during the window of implantation. A comprehensive understanding of the molecular and cellular factors governing this process is crucial for deciphering the complexities of fertility and devising targeted interventions for individuals facing challenges in conception. Despite its known presence and significance, CD-138 remains enigmatic in many aspects. The existing body of literature provides glimpses into its potential roles, yet a comprehensive understanding needs to be improved by significant knowledge gaps. Unraveling the mysteries surrounding CD-138 could shed light on its involvement in infertility and early pregnancy, providing a basis for developing novel diagnostic and therapeutic strategies.

## Review

Endometrial CD-138: an overview

Molecular Structure and Function

Description of CD-138 structure: CD-138, syndecan-1, is a protein evaluated in endometrial biopsies for detecting subclinical chronic endometritis. This condition can disrupt the uterine environment and is crucial for successful embryo implantation. Research indicates that individuals experiencing recurrent implantation failure often exhibit an elevated incidence of chronic endometritis, as indicated by a positive CD-138 result. It is worth noting that endometrial plasma cells are present in approximately half of the general infertile population. However, their presence alone does not serve as a reliable predictor for clinical pregnancy, clinical pregnancy loss, or live birth rates at lower levels [4-9]. The available search results did not yield specific information for an in-depth exploration of the molecular structure and function of CD-138. In that case, a more comprehensive understanding of the molecular intricacies and functional aspects of CD-138 within the context of endometrial health and infertility may be obtained.

Physiological functions in the endometrium: The endometrium, an intricate and dynamic multicellular tissue, exhibits responsiveness to ovarian hormones. Its physiological functions encompass preparing for implantation, sustaining pregnancy post-implantation, and facilitating menstruation without pregnancy. Undoubtedly, the endometrium assumes a pivotal role in the reproduction and perpetuation of our species. The interplay between the hypothalamus, pituitary gland, and ovaries intricately regulates the cyclic transformations of human endometrial cells. This renders the endometrium remarkably dynamic and highly adaptable to fluctuations in circulating levels of sex hormones. The endometrium undergoes successive exposures throughout the menstrual cycle, initially to systemic estradiol, followed by concurrent exposure to estradiol and progesterone, and ultimately, progesterone withdrawal in the absence of pregnancy. This sequential exposure prompts the endometrium to adapt in form and function across the menstrual cycle. Noteworthy characteristics of the endometrium involve rapid repair processes that occur without leaving residual scarring or loss, akin to maintaining a fetus in utero. The menstrual endometrium is a physiological exemplar of a surface experiencing injury or "wounding" that undergoes repair without resulting in scarring. The physiological unfolding of menstruation and endometrial repair offers a tangible in vivo human model for studying tissue injury and repair processes [10-13].

Cellular Localization

Distribution of CD-138 in different endometrial cell types: The presence and distribution of CD-138 across various types of endometrial cells are predominantly associated with its diagnostic significance in chronic endometritis (CE) and its potential repercussions on pregnancy outcomes. CD-138, syndecan-1, is a transmembrane heparan sulfate proteoglycan that functions as an extracellular matrix receptor and is a specific marker for plasma cells. In the context of endometrial health and infertility, identifying CD-138+ cells within the endometrial stroma has been correlated with the diagnosis of CE and its potential impact on pregnancy outcomes [14]. Studies have revealed that CD-138+ cells, particularly in the proliferative-phase endometrium, may be an adverse indicator for pregnancy outcomes in in vitro fertilization (IVF) cycles. Elevated counts of CD-138+ cells in the endometrium have been linked to pregnancy failure and are proposed to possess a specific predictive value for pregnancy outcomes [15,16]. Moreover, CD-138 immunohistochemistry has been employed to enhance the diagnostic accuracy of CE. Research indicates that the quantification of CD-138-positive cells can be a reliable method for CE detection, with a diagnostic criterion of five or more CD-138+ cells per high-power field (HPF) [4,8,9,15].

Changes in expression levels across the menstrual cycle: Research has explored the distribution of CD-138 within the endometrium and its correlation with CE and pregnancy outcomes. CD-138 serves as a marker for plasma cells, and its presence in the endometrial stroma has been associated with the diagnosis of CE and its potential influence on pregnancy outcomes. Elevated counts of CD-138+ cells in the endometrium have been linked to pregnancy failure and are considered to have a specific predictive value for pregnancy outcomes [6,9,17]. Furthermore, CD-138 immunohistochemistry has been employed to enhance the diagnostic accuracy of CE. Studies suggest that the quantity of CD-138-positive cells can serve as a reliable method for detecting CE, with a diagnostic criterion of five or more CD-138+ cells per HPF [9,17]. Although there is limited information on the variations in CD-138 expression levels throughout the menstrual cycle, its presence in the endometrial stroma has been associated with the diagnosis of CE and its potential impact on pregnancy outcomes. CD-138 immunohistochemistry has proven valuable in improving the diagnosis rate of CE, with a higher number of CD-138-positive cells being linked to pregnancy failure.

CD-138 in infertility

Studies Linking CD-138 and Infertility

Review of clinical studies and observations: Numerous studies have established a connection between CD-138, infertility, and pregnancy outcomes. The count of CD-138 in the endometrium appears to serve as a negative prognostic indicator, particularly for patients who have faced previous embryo transfer failures. A higher expression of CD-138-positive cells correlates with poorer pregnancy outcomes. Moreover, free pelvic fluid on the day of endometrial sampling has been identified as a potential risk factor for CD-138 expression [6]. In patients not expressing CD-138, there were statistically significantly higher clinical pregnancy and embryo implantation rates [6]. Chronic endometritis, diagnosed through endometrial biopsy and the analysis of plasma cells, has been implicated as a contributing factor to implantation failure [5]. CD-138 immunohistochemistry has proven effective in enhancing CE diagnostic accuracy [4,9]. The presence of CD-138 in the endometrium has been associated with adverse pregnancy outcomes, particularly in cases of prior embryo transfer failure. Additionally, CD-138 has been implicated in the diagnosis of chronic endometritis, which can compromise endometrial receptivity and contribute to infertility or miscarriage. The utilization of CD-138 immunohistochemistry emerged as a reliable method for detecting chronic endometritis, offering potential implications for the management of infertility and assisted conception treatments. Numerous clinical studies and observations have explored the relationship between CD-138 expression and infertility. The count of CD-138 in the endometrium emerges as a negative prognostic indicator for patients with a history of previous embryo transfer failure. A higher number of CD-138+ cells is associated with poorer pregnancy outcomes.

Mechanisms Implicated in Infertility

Male infertility: The molecular mechanisms regulating male fertility have been subject to research, revealing species-dependent pathways that play a crucial role in the fertility of mammalian sperm. Key factors identified in this context include mitochondrial-associated signaling pathways and the accurate synthesis and folding of proteins during spermatogenesis. Furthermore, comprehensive omics studies, encompassing analysis of transcript, protein, and metabolite levels, have significantly contributed to advancing our understanding of male fertility potential [18].

Female infertility: Female infertility has been linked to various factors, including genetic abnormalities, hormonal signaling deficiencies, and conditions such as endometriosis. Genetic factors are critical in essential processes like oocyte maturation, fertilization competence, and preimplantation development. Deficiencies in hormonal signaling, especially those related to gonadotropins, can impact folliculogenesis, oocyte maturation, and the implantation process [19].

Endometriosis-associated infertility: Endometriosis can have a detrimental impact on the interaction between sperm and the endosalpinx epithelium. Molecular pathways associated with endometriosis-related infertility encompass the Ras signaling pathway, cAMP signaling pathway, and ovarian steroidogenesis. Additionally, abnormal amino acid metabolism and pathways related to nicotine and cocaine addiction have been implicated in the context of endometriosis-associated infertility [20,21].

Implantation failure: Molecular mechanisms crucial for implantation, including adhesion and tissue remodeling, are pivotal in establishing successful pregnancies. Factors such as mechanical interference with sperm transport, altered expression of specific proteins and transcription factors, and uterine receptivity can significantly influence the success of implantation [22]. The implicated molecular pathways in infertility are diverse, covering genetic, endocrine, and physiological factors. They also include specific mechanisms related to male and female infertility, endometriosis, and implantation failure. A comprehensive understanding of these pathways is essential for the accurate diagnosis and effective management of infertility.

Insights From Animal Models and In Vitro Studies

Disease modeling is a pivotal component of biomedical research, employing animal models, especially genetically engineered ones, to deepen our understanding of human disease pathogenesis and propel advancements in treatment development. Notably, large animal models have played a crucial role in the exploration of neurodegenerative diseases such as Alzheimer's and Parkinson's [23,24]. In infection prevention and treatment, in vitro studies assess the effectiveness of antimicrobial coatings, percutaneous implants, and other anti-infective interventions [25]. Additionally, genetically modified animal models, including pigs and non-human primates, significantly contribute to evaluating gene therapies and drug safety in the context of gene therapy and drug development [23,24].

In the pursuit of comprehending neuronal regeneration and repair, in vitro studies focusing on stem cells and their interactions with the extracellular matrix provide valuable insights into the potential for addressing neurological disorders [26]. Similarly, investigations into immune system function, involving in vitro studies on immune cells and their interactions with various cell types, assist researchers in understanding the immune response and developing therapies for autoimmune and immune-related disorders [27]. Despite their significance, both animal models and in vitro studies face challenges such as the high cost associated with large animal models, the inefficiency of gene targeting in larger animals, and the time-intensive nature of these studies [23]. Nevertheless, these methodologies remain indispensable in biomedical research, offering essential insights into the mechanisms of human diseases and potential therapeutic interventions [28,29].

CD-138 in early pregnancy

Expression Patterns During Implantation

Examination of CD-138 expression during the implantation window: CD-138, also known as syndecan-1, is a protein with implications for the immune system, and its role in early pregnancy and implantation has been a subject of study. During the proliferative phase of the endometrium, the presence of CD-138+ cells has been identified as an adverse indicator for pregnancy outcomes in fresh IVF/intracytoplasmic sperm injection (ICSI) cycles, with a specific quantitative threshold [16]. Although endometrial plasma cells, which express CD-138, are present in half of the general infertile population, they do not serve as predictive markers for clinical pregnancy, clinical pregnancy loss, or live birth rates when present at low levels [4].

CD-138 immunohistochemistry proves valuable in enhancing the diagnostic accuracy of chronic endometritis, a condition that has the potential to dysregulate the uterine environment crucial for embryo implantation [9]. Individuals with a previous history of prolonged menstrual bleeding episodes, a record of abortion, and a history of fallopian tube obstruction are identified as being at risk for chronic endometritis, warranting a recommendation for a CD-138 immunohistochemical examination in such cases [9]. The expression patterns of CD-138 during implantation offer valuable insights into pregnancy outcomes and the presence of chronic endometritis. However, it is noteworthy that further investigation is required to ascertain its predictive value and assess its impact on early pregnancy.

Interaction with embryonic tissues: CD-138, also known as syndecan-1, is a protein with a pivotal role in the immune system, and its potential impact on early pregnancy and implantation has been a subject of study. Its expression is predominantly found on mature plasma cells and early preB-cells, while other hematolymphoid cells do not exhibit CD-138 expression [30]. CD-138 plays a facilitating role in transepithelial leukocyte migration and in regulating IL-8 and IT [30]. Moreover, CD-138 expression is essential for Wnt-1-induced tumorigenesis in mice [30]. Functionally, CD-138 serves as a crucial regulator of cell-cell and cell-extracellular matrix interactions. Under experimental conditions, the loss of CD-138 expression induces changes in the morphology of normal mammary epithelial cells. Conversely, induced protein expression has the opposite effect on cell morphology [30]. These findings underscore the multifaceted roles of CD-138 in immune function, cellular interactions, and its potential implications in tumorigenesis. The over-expression of CD-138 has been found to inhibit the growth and migration of affected cells [30]. The predictive value of CD-138 expression in anticipating tumor behavior varies across tumor types and differentiation levels. In squamous cell carcinogenesis, there is reduced expression, while in renal cell carcinomas, low-level expression correlates with increasing nuclear grade. Conversely, some cases exhibit a correlation between the over-expression of CD-138 [30].

Role in Trophoblast-Endometrial Cross-Talk

Insights into the communication between trophoblasts and the endometrium: During the early stages of pregnancy, notable morphological changes occur in both the embryo and the endometrium, marking a crucial communication period between the maternal uterus and blastocyst. Trophoblast cells play a key role in this communication, releasing factors such as chorionic gonadotropin (CG) and extracellular vesicles (EVs) at an early stage of embryo development. These secreted elements can induce unique transcriptomic alterations in target endometrial cells [31-33]. The interaction between trophoblasts and the endometrium is important for successful embryo implantation. Both trophoblast and endometrium are involved in balancing the expression of growth factors, cytokines, and enzymes to regulate the extent of trophoblast invasion [33]. The molecular cross-talk between the developing embryo and the endometrium is intricate, encompassing various signaling pathways that govern trophoblast and endometrial functions [34,35]. While the underlying signaling mechanisms remain fully elucidated, trophoblast-endometrial cross-talk is considered critical for achieving implantation and pregnancy.

Implications for successful implantation: Embryo implantation success is influenced by multiple factors, including the embryo's health, receptivity of the endometrium, and the individual's systemic health. Factors affecting implantation encompass thyroid health, blood sugar levels, smoking, alcohol consumption, and weight [36]. In mice, uterine sensitivity to implantation is categorized into three principal phases: pre-receptive, receptive, and nonreceptive [37]. In humans, three primary factors contribute to embryo implantation: quality, endometrial receptivity, and the technique used for embryo transfer [38]. The successful implantation process necessitates a well-coordinated interaction between the developing embryo and the endometrium, involving various molecular and hormonal signaling pathways [39]. The study of endometrial CD-138 count has been explored as a potential marker that may predict pregnancy outcomes following embryo transfer, suggesting its potential role in the success of implantation [6]. The quality of embryo implantation has been demonstrated to determine the quality of ongoing pregnancy and fetal development [40]. In summary, a combination of factors related to both the embryo and the maternal environment plays a crucial role in the successful implantation of an embryo.

Regulation of CD-138 expression

Hormonal Regulation

Influence of estrogen and progesterone on CD-138 expression: CD-138, syndecan-1, is a transmembrane protein with roles in cell migration and cytoskeletal organization [41]. Its expression undergoes alterations in various cancer types and is linked to unfavorable clinical outcomes [41]. Several factors, including hormones such as estrogen and progesterone, play a role in regulating CD-138 expression. In breast cancer, the staining intensity of CD-138 was not influenced by estrogen receptor (ER) expression, while progesterone receptor (PR) expression significantly affected the intensity [42]. In mouse endometrial stromal cells, progesterone partially reduced the increase in CD-138 expression induced by co-culture with endometrial stromal cells [43]. However, the specific function of CD-138 in endometrial stromal cells and its regulation by hormones require further investigation. CD-138 expression is under hormonal regulation, involving estrogen and progesterone, with varying effects on cancer types and cells. Additional research is necessary to comprehensively understand the role of CD-138 in the context of endometrial stromal cells and its potential implications for implantation and pregnancy outcomes.

Feedback mechanisms in the endometrium: Feedback mechanisms within the endometrium play a pivotal role in sustaining hormonal equilibrium and facilitating successful embryo implantation. These mechanisms entail intricate interactions among hormones, notably estrogen, progesterone, and endometrial tissue. A predominant negative feedback system comes into play towards the end of the menstrual cycle, primarily under the influence of progesterone. This system prevents premature development of the corpus luteum and fosters the growth of the endometrial lining [44]. In preparation for embryo implantation, the endometrium undergoes crucial changes, including an increase in vascular supply and stimulation of mucous secretions. Progesterone, in particular, prompts the endometrium to slow the reduction of lining thickness, form more complex glands, accumulate energy sources like glycogen, and enhance surface area within the spiral arteries [44]. The human endometrium exhibits remarkable plasticity, evident through rapid growth and differentiation throughout the menstrual cycle, accompanied by swift tissue turnover. Mechanical forces contribute to these processes, with the sensing and response to such forces being essential for endometrial receptivity and successful implantation [45]. Trophoblast-endometrial communication, specifically through trophoblast-derived EVs, can modify endometrial gene expression, potentially holding functional significance in embryo-maternal communication during implantation [31]. Disturbances in these intricate feedback mechanisms may occur due to increased ovarian activity, potentially leading to menstrual irregularities and other reproductive issues [46]. Overall, feedback mechanisms in the endometrium are essential for maintaining hormonal balance and ensuring the successful preparation of the endometrium for implantation, involving a complex interplay of hormones, mechanical forces, and trophoblast-endometrial communication.

Other Regulatory Factors

Inflammatory mediators and cytokines in regulating the endometrium, inflammatory mediators, and cytokines play a significant role in orchestrating the immune response and facilitating tissue remodeling. The endometrium undergoes dynamic changes in response to these factors to support the processes of embryo implantation and pregnancy. CD-138 is intricately associated with inflammation and immune responses. Elevated soluble CD-138 expression has been correlated with inflammation and its potential to modulate the activity of cytokines and growth factors [47,48]. The immune function of the endometrium is intricately regulated by hormonal changes, particularly those involving estrogen and progesterone. The temporal coordination between the immune and endocrine networks is critical in establishing immunological tolerance at the onset of implantation [49]. This interplay between inflammatory mediators, cytokines, and hormonal regulation underscores the intricate mechanisms involved in the endometrial preparation for embryo implantation and the maintenance of pregnancy. The intricate interplay between inflammatory mediators, cytokines, and hormonal regulation is critical in shaping the endometrium's immune function and facilitating tissue remodeling. Ultimately, these factors collectively influence the success of embryo implantation and the progression of pregnancy [44]. Genetic and epigenetic factors intricately govern the expression of CD-138, a transmembrane protein recognized for its involvement in cell migration and cytoskeletal organization. The impact of these factors extends across a spectrum of physiological and pathological processes, notably influencing cancer, immune function, and the survival of plasma cells [48].

Genetic factors are nuanced in CD-138 expression, as evidenced by its presence on a specific subset of thymic inducible natural killer T (iNKT) cells, particularly the RORγt+ NKT17 cells [48]. Notably, the absence of CD-138 does not hinder the development of NKT17 cells, implying that CD-138 is not a prerequisite for generating these cells [48]. On the epigenetic front, the interplay between genetic and epigenetic factors emerges as a critical determinant of CD-138 expression. In the context of plasma cell differentiation, multiple cell division-coupled epigenetic programs regulate CD-138 expression [50]. Moreover, in the realms of multiple myeloma and breast cancer, CD-138's significance is underscored by its binding to the survival factor APRIL, and it acts as a co-receptor for growth factors such as hepatocyte growth factor and epidermal growth factor, thereby promoting cell survival [47].

Cytokine regulation further amplifies the role of CD-138, as it can bind to and modulate the activity of soluble factors, including cytokines and growth factors [41]. Notably, elevated soluble CD-138 expression has been associated with inflammatory processes and heightened leukocyte responses [41]. The nexus between CD-138, cancer, and immune function is profound. Altered CD-138 expression has been documented across various neoplasias, encompassing cancers of the breast, urinary bladder, pancreas, ovary, and endometrium [41]. Strikingly, in some tumor contexts, fluctuations in CD-138 expression levels have been linked to unfavorable tumor phenotypes [41]. The intricate regulation of CD-138 by genetic and epigenetic factors underscores its pivotal role in diverse physiological and pathological processes, including immune function, plasma cell survival, and cancer. This multifaceted involvement positions CD-138 as a key player in the intricate network orchestrating cellular dynamics in health and disease.

Diagnostic and therapeutic implications

CD-138 as a Diagnostic Marker

CD-138 emerges as a promising diagnostic marker pertinent to infertility and pregnancy complications, particularly in the context of endometrial biopsy for CE [6,8,9]. The presence of CD-138 in the endometrium serves as a negative prognostic indicator, particularly for individuals with a history of previous embryo transfer failures [6,8]. Furthermore, CD-138 immunohistochemistry proves valuable in augmenting the diagnostic accuracy of chronic endometritis, a condition known to dysregulate the uterine environment and is crucial for successful embryo implantation [9]. Risk factors such as a history of prolonged menstrual bleeding episodes, abortion, and fallopian tube obstruction underscore the importance of recommending CD-138 immunohistochemical examinations in individuals with such medical histories [9]. While chronic endometritis diagnosed through endometrial biopsy and CD-138 analysis has been identified as a potential cause of implantation failure, the specific endometrial staining for CD-138 as a marker does not independently predict failed implantation. This suggests that other factors may be more significant in implantation failure [5]. The diagnostic potential of CD-138 in infertility and pregnancy complications, particularly in the context of chronic endometritis, is evident. However, further investigations are warranted to comprehensively understand its diagnostic and therapeutic implications in the intricate processes of the uterine environment and embryo implantation.

Diagnostic Assays and Techniques

CD-138 serves as a pivotal diagnostic marker, indicating the presence of plasma and squamous epithelial cells, and plays a crucial role in diagnosing chronic endometritis and plasma cell neoplasms [51,52]. The diagnostic process involves immunohistochemistry (IHC) on formalin-fixed, paraffin-embedded tissue samples. The technical performance and subsequent interpretation of the stain are typically conducted by a pathologist within the context of the patient's clinical history [51,52]. The preferred specimen type for optimal testing is a formalin-fixed, paraffin-embedded (FFPE) tissue block, with additional requirements including unbaked, unstained slides [51]. The detection methodology specifically employs IHC, utilizing specific antibodies to visualize the presence of the CD-138 protein within the tissue sample [51,52]. The clinical application of CD-138 extends to diagnosing various conditions, encompassing plasma cell neoplasms and chronic endometritis. The versatility of CD-138's expression across different cell types and its specific role in identifying distinct diseases underscores its significance as a valuable diagnostic tool in clinical practice [51,52]. CD-138, detected through immunohistochemistry, emerges as a crucial diagnostic marker with diverse clinical applications, contributing significantly to the accurate diagnosis of various medical conditions.

Therapeutic Approaches Targeting CD-138

Anti-CD-138-targeted interferon, specifically anti-CD-138-IFNα2, has demonstrated increased potency compared to untargeted anti-CD20 when directed at multiple myeloma cells [53]. Exploration of therapeutic strategies targeting CD-138 extends to chimeric antigen receptor (CAR) T-cell therapy. CAR-T cells designed to target CD-138 have shown substantial efficacy in preclinical studies, with an ongoing clinical trial actively enrolling participants [54]. CD-138 has been evaluated as a target for addressing multiple myeloma in nanoparticle-based drug delivery. Interestingly, CD-138-targeted nanoparticles, while explored, were found to be less effective than their CD-138-targeted counterparts in in vivo studies [55]. Monoclonal antibody therapy presents a novel avenue with developing VIS832, an anti-CD-138 monoclonal antibody. VIS832 has demonstrated the ability to induce the killing of multiple myeloma cells in humans both in vitro and in vivo, exhibiting enhanced efficacy when combined with lenalidomide or bortezomib [56]. Adoptive immunotherapy enters the therapeutic landscape with T cells expressing a tumor-associated CAR targeting CD-138. This approach holds promise for multiple myeloma therapy, further emphasizing CD-138 as a potential therapeutic target in multiple myeloma and other medical conditions [57].

Challenges and Future Directions

Challenges in diagnosing chronic endometritis persist despite the advancements brought about by CD-138 immunohistochemistry. While this method has improved diagnosis rates, the absence of standardized diagnostic criteria and methods remains a hurdle. Moreover, the subjective interpretation of CD-138 staining poses a challenge, introducing variability in results between pathologists. On the therapeutic front, CD-138 has been identified as a potential target in treating multiple myeloma. However, the development of effective therapies faces challenges, exemplified by the comparatively lower efficacy of CD-138-targeted nanoparticles in vivo compared to CD-138-targeted counterparts. Additionally, a dearth of clinical trials evaluating the safety and efficacy of CD-138-targeted therapies across various medical contexts complicates the translation of this potential target into clinical applications.

Looking ahead in the realm of diagnosis, future research avenues may concentrate on developing more specific and sensitive diagnostic assays for CD-138. Promisingly, the use of flow cytometry to detect CD-138 expression in plasma cells has shown potential as a diagnostic tool, particularly in the context of multiple myeloma. In the arena of therapy, future research directions could explore the development of combination therapies that target CD-138 alongside other proteins or pathways implicated in disease progression. The advent of CAR T-cell therapy targeting CD-138 emerges as a particularly promising area of research, potentially unlocking more effective treatments for multiple myeloma. Despite the promise shown by CD-138 as a diagnostic marker and therapeutic target, the road ahead involves addressing challenges through standardized diagnostic criteria, pursuing more effective therapies, and exploring combination therapies and innovative immunotherapeutic approaches such as CAR T-cell therapy. These directions underscore the dynamic nature of ongoing research aimed at enhancing the clinical utility of CD-138 in various medical contexts.

## Conclusions

In conclusion, this comprehensive review has provided a nuanced understanding of the pivotal role played by CD-138 in the endometrium, shedding light on its significance in infertility and early pregnancy. The intricate dance orchestrated by CD-138 within the endometrial microenvironment, influencing factors crucial for fertility and successful embryo implantation, has been unveiled through a synthesis of key findings. The implications for clinical practice are profound, with CD-138 emerging as a potential diagnostic marker for assessing endometrial receptivity and guiding personalized treatment strategies. The integration of CD-138 assessments into existing fertility diagnostics holds promise for refining prognostic accuracy, particularly in assisted reproductive technologies. Looking forward, the identified knowledge gaps beckon future research endeavors, urging exploration into the regulatory networks governing CD-138 expression. The prospect of harnessing this newfound understanding of precision medicine in reproductive health underscores the transformative potential of CD-138 in reshaping the landscape of fertility interventions as we navigate the evolving narrative of endometrial CD-138, the promise of improved clinical outcomes and a deeper comprehension of reproductive biology beckons on the horizon.
